# Skeletal Anomalies in The Neandertal Family of El Sidrón (Spain) Support A Role of Inbreeding in Neandertal Extinction

**DOI:** 10.1038/s41598-019-38571-1

**Published:** 2019-02-08

**Authors:** L. Ríos, T. L. Kivell, C. Lalueza-Fox, A. Estalrrich, A. García-Tabernero, R. Huguet, Y. Quintino, M. de la Rasilla, A. Rosas

**Affiliations:** 1 Department of Physical Anthropology, Aranzadi Zientzia Elkartea, Zorroagagaina 11, 20014 Donostia, Gipuzkoa, Basque Country Spain; 2 Skeletal Biology Research Centre, School of Anthropology and Conservation, University of Kent, Marlowe Building, Canterbury, CT2 7NR UK; 3 Department of Human Evolution, Max Planck Institute for Evolutionary Anthropology, Deutscher Platz 6, Leipzig, 04103 Germany; 4 Institute of Evolutionary Biology (CSIC–Universitat Pompeu Fabra), Carrer Dr. Aiguader 88, 08003 Barcelona, Spain; 5 Instituto Internacional de Investigaciones Prehistóricas de Cantabria IIIPC (Universidad de Cantabria, Santander, Gobierno de Cantabria), Avda. de los Castros 52, 39005 Santander, Cantabria Spain; 6 Paleoanthropology Group, Department of Paleobiology. Museo Nacional de Ciencias Naturales (MNCN-CSIC), José Gutiérrez Abascal 2, 28006 Madrid, Spain; 7 IPHES, Institut Catala de Paleoecologia Humana i Evolució Social, Campus Sescelades URV (Edifici W3), 43007 Tarragona, Spain; 8 Area de Prehistoria, Universitat Rovira i Virgili, Avda. Catalunya 35, 43002 Tarragona, Spain; 9 Unidad asociada al CSIC, Departamento de Paleobiología, Museo Nacional de Ciencias Naturales, Calle José Gutierrez Abascal 2, 28006 Madrid, Spain; 10 Laboratorio de Evolución Humana, Dpto. de Ciencias Históricas y Geografía, Universidad de Burgos, Edificio I+D+i, Plaza Misael Bañuelos s/n, 09001 Burgos, Spain; 11 Área de Prehistoria Departamento de Historia, Universidad de Oviedo, Calle Teniente Alfonso Martínez s/n, 33011 Oviedo, Spain

## Abstract

Neandertals disappeared from the fossil record around 40,000 bp, after a demographic history of small and isolated groups with high but variable levels of inbreeding, and episodes of interbreeding with other Paleolithic hominins. It is reasonable to expect that high levels of endogamy could be expressed in the skeleton of at least some Neandertal groups. Genetic studies indicate that the 13 individuals from the site of El Sidrón, Spain, dated around 49,000 bp, constituted a closely related kin group, making these Neandertals an appropriate case study for the observation of skeletal signs of inbreeding. We present the complete study of the 1674 identified skeletal specimens from El Sidrón. Altogether, 17 congenital anomalies were observed (narrowing of the internal nasal fossa, retained deciduous canine, clefts of the first cervical vertebra, unilateral hypoplasia of the second cervical vertebra, clefting of the twelfth thoracic vertebra, diminutive thoracic or lumbar rib, os centrale carpi and bipartite scaphoid, tripartite patella, left foot anomaly and cuboid-navicular coalition), with at least four individuals presenting congenital conditions (clefts of the first cervical vertebra). At 49,000 years ago, the Neandertals from El Sidrón, with genetic and skeletal evidence of inbreeding, could be representative of the beginning of the demographic collapse of this hominin phenotype.

## Introduction

The causes of the extinction of the Neandertal populations in western Eurasia by 40,000 BP^1^ is a topic of intense debate in human evolution. Some interpretations attribute this extinction to competition with early anatomical modern humans (AMHs), which would present differences expressed for instance through more efficient exploitation of dietary resources, possibly related to differential cognitive, behavioral and cultural abilities, that could rest on life-history and ontogenetic differences^2–5^. However, other interpretations have recognized recent findings that support Neandertal dietary flexibility and multiple subsistence strategies^6,7^, increasing evidence of symbolic behavior and complex technologies^8–13^, and lack of fundamental differences in the overall pace of dental and skeletal growth and maturation in comparison with AMHs^14^, all of which complicate a scenario of AMHs simply outcompeting Neandertals^15^. Environmental change also has been considered as a potential important factor in the Neandertal demise, whether acting independently, or in combination with other previously-mentioned differences between the two hominins^16–18^. In the context of competition, the very fact of interbreeding within what has been called a hominin metapopulation^19^, would suggest a complex interaction between Neandertal, AMHs populations and Denisovans that has yet to be defined in detail^20^. For instance, demographic and ecocultural modeling have included competition models, based on cultural and demographic differences^21,22^, and selectively-neutral models, based on migration dynamics and local dispersal and replacement alone (in absence of culturally-driven selection or environmental factors)^23^, with both resulting in the replacement of Neandertals by AMHs. Other models conclude that hunting-prey decline or climatic variations alone was not sufficient to cause the disappearance of Neandertals^24^. In all cases, most researchers agree that, “whatever the extent to which the eventual replacement of late archaic human morphology involved admixture, absorption, and/or population displacement, the process was ultimately a demographic one”^25^.

In this regard, it has been suggested that the archaeological evidence supports substantial demographic differences at the Neandertal-to-AMH transition, with up to a tenfold increase in population density for early AMHs compared with Neandertals^26^, that could have been a critical factor in the Neandertal demise. Although others have recommended caution when making inferences about population size from the archaeological record^27^, it is not unreasonable to suggest that demographic differences in population size and density, and in group size, could have been an important factor in the disappearance of Neandertals^28^. In addition, the general demographic structure of Pleistocene *Homo*, with small effective population sizes (see below), a hunter-gatherer existence and population dispersal into separate small kindred groups, would have favored substantial levels of intragroup, and potentially intrafamily, mating^29,30^. Important contributions to Neandertal paleodemography in this direction come from genetic studies, where high levels of inbreeding, or mating among relatives, and a general decrease in heterozygosity have been observed. Specifically, Neandertals from the Altai, Vindija, Mezmaiskaya and El Sidrón sites present low levels of heterozygosity and small estimated effective population sizes averaging around 3000 individuals, both characteristics considered typical of archaic hominins, indicating that they lived in small and isolated populations^31^. Studies of genetic homozygosity indicate that Neandertals had a long history of high but variable levels of inbreeding. The most extreme values are found in the Altai Neandertal, with long stretches of homozygosity that indicate recent inbreeding consistent with parental relatedness between two half-siblings^31^. In contrast, Vindija Neandertal homozygosity is comparable to modern human groups like the Karitiana and Pima, suggesting that consanguinity was not ubiquitous among all Neandertal populations^31^. At El Sidrón, a Neandertal sample (SD1253) had a larger cumulative length of homozygous genomic stretches of 10–100 Kb than samples from Vindija, Altai, Denisova, great apes and modern humans^32^, indicating a long history of inbreeding. In addition, the mitochondrial DNA (mtDNA) analysis of twelve El Sidrón individuals revealed low mtDNA genetic diversity and close kin relationships within the group^33^.

Within this context, it is reasonable to expect that a scenario of small, isolated groups of Pleistocene *Homo* with potentially high levels of intragroup mating would be also phenotypically expressed in the skeleton. For instance, recent analyses of bony labyrinth morphology in the Aroeira 3 cranium suggest a degree of demographic isolation in geographically and chronologically close hominins around the origin of the Neandertal clade^34^, and as previously suggested^35^ and recently shown^36^, there is a high incidence of developmental abnormalities and anomalies in Pleistocene *Homo*, several of them very rare or with unknown etiology. In past and present modern human populations, dental and skeletal anomalies and low-frequency anatomical variants have been associated with geographical isolation and/or endogamy^37^. Given the nuclear and mtDNA genetic evidence that indicates that the 13 individuals from El Sidrón constitute a closely related kin group^33^, El Sidrón is the ideal Pleistocene sample to test for skeletal evidence of inbreeding. Previous morphological analyses of the El Sidrón Neandertals have reported congenital clefts of the first cervical vertebra^37^ and the retention of a deciduous mandibular canine in two individuals^38^, but a systematic analysis of the entire sample has not yet been done. Here we present the results of the complete morphological analysis of the 1674 identified skeletal specimens from a total of 2556 remains recovered from El Sidrón.

## Results

We define anomalies as bone variants, both pathological and non-pathological, deviating from normal structure^39–42^ (Supplementary Information SI1). Our objective, rather than to obtain a differential diagnosis, was to state that the anomalies were congenital, a term understood as a condition that is present at birth and genetically driven, after discarding alternative explanations such as traumatic and infectious conditions, environmental stress and taphonomic processes as the cause of the observed anomalies (Supplementary Information SI1).

### Maxilla and Mandible

**Figure 1:**
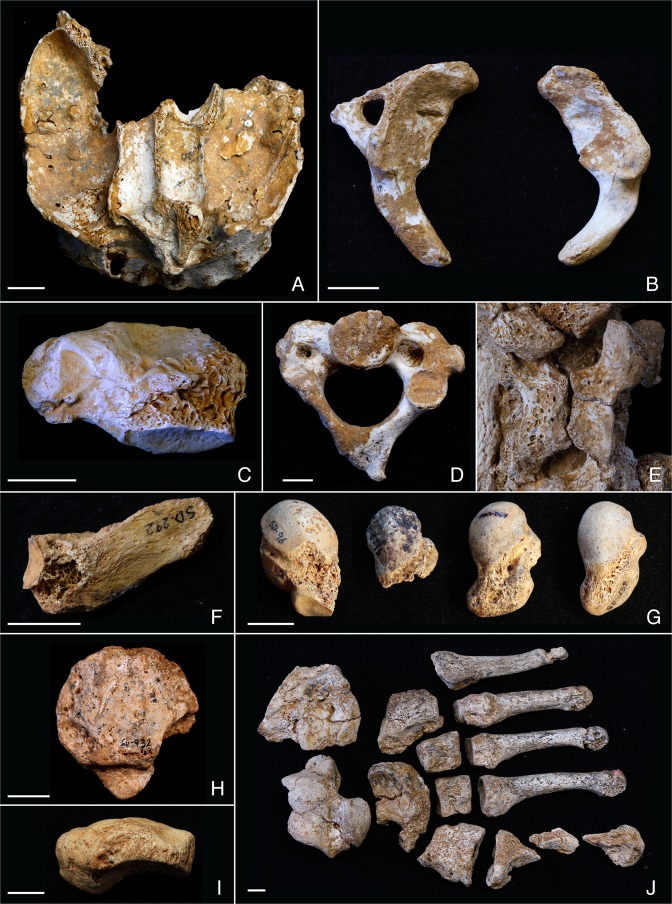
Bones with congenital anomalies within the El Sidrón family group. Maxilla (**A**), first cervical vertebrae (**B**,**C**), second cervical vertebra (**D**), twelfth thoracic vertebra (**E**), twelfth hypoplastic rib or lumbar rib (**F**), os centrale and bipartite scaphoid (**G**), tripartite patella (**H**), navicular-cuboid non-osseous coalition (**I**), left foot anomaly (**J**).

The El Sidrón Adult 2 (A2) preserves several morphological features of interest in its maxilla and mandible (Fig. 1A). As described previously^38^, this individual, as well as El Sidrón Adolescent 3, retains a left mandibular deciduous canine. A metric comparison of the internal nasal fossa breadth of El Sidrón A2 (22.47 mm) with other Neandertals (mean 34.13 mm)^43^ and with modern humans (overall mean 32.87 mm, Arctic population mean 30.3 mm)^44^, places this maxilla at the smallest extreme of the observed range of variation (Supplementary Information SI2, Supplementary Fig. S1). The maxilla also has a right-side deviation of the anterior nasal crest along its entire length, and asymmetry to the dental arcade, although interpretation of both of these anomalies is hampered by taphonomic alteration of the bone (Supplementary Fig. S1). Our interpretation is that, besides the retained deciduous canine, the narrowing of the internal nasal fossa would be consistent with a congenital condition. A narrowing of the internal nasal fossa is present in several conditions, from congenital nasal pyriform aperture stenosis^45–47^, a condition potentially related to other anomalies and occurring in modern humans in approximately 1 in 25,000 births^48^, to more complex conditions affecting the middle third of the face, such as Goldenhar^49^, Aper^50^ and Binder^51^ syndromes. Since no other clear anomalies were observed in this anatomical region, complex conditions similar to the latter ones are not likely.

### Vertebrae and Ribs

Several El Sidrón individuals preserve evidence of sagittal clefts of the cervical vertebrae. A first cervical vertebra (C1 or atlas) fragment (SD-636) shows evidence of a congenital anterior sagittal cleft (Fig. 1B, Supplementary Information SI3, Supplementary Fig. S2), similar to another C1 fragment (SD-1094) recently described^37^ as having another congenital anterior sagittal cleft. In addition, an almost complete atlas from El Sidrón (SD-1643) preserves a congenital posterior sagittal cleft^37^ and there are two C1 hemi-arches (SD-2045 and SD-1725) from the juvenile skeleton El Sidrón J1 interpreted as a congenital posterior sagittal cleft^14^. In modern humans, the posterior synchondrosis is fused by six years of age in 95% of individuals and the remaining 5% correspond to cases of congenital posterior sagittal clefts^37^. Thus the lack of fusion of the posterior synchondrosis PS in El Sidrón J1 is best interpreted as a congenital posterior sagittal cleft of the C1^14,37^. In total, four out of five atlas specimens with observable anterior or posterior sagittal arches and four out of 13 identified Neandertal individuals at El Sidrón present congenital clefts of the atlas. The frequency of atlas congenital clefts in modern humans ranges from 0.087% to 0.1% for the anterior cleft, and from 0.73% to 3.84% for the posterior cleft^37^. These clefts have been associated with several different congenital conditions, including Down’s syndrome, Chiari malformation, Klippel-Feil, Goldenhar syndrome, Conradi syndrome, and Loeys-Dietz syndrome, where a higher frequency of anterior (24%) than posterior (16%) C1 clefts have been observed^37,52^. It is important to stress, however, that in modern humans these clefts are usually asymptomatic and often only identified in routine examinations^37^.

A second cervical vertebra (C2 or axis) (SD-1601) preserves several morphological features consistent with congenital alterations. First, the right transverse process has not developed and there is bilateral asymmetry in the size of the transverse foramina (Fig. 1C, Supplementary Information SI4, Supplementary Fig. S3). The vertical/horizontal and transverse/longitudinal diameters of the right transverse foramen at its lateral and inferior borders fall at the smallest extreme of the modern human range of variation, while the diameters for the left foramen fall well within this interval^53,54^ (Supplementary Information SI4, Supplementary Tables S1,S2). Second, the right half of the tip of the spinous process has also not developed and there is bilateral asymmetry in the thickness of the laminae (Fig. 1C, Supplementary Fig. S3). The value of the thickness of the right lamina falls at the smallest extreme of modern human variation, while the thickness of the left lamina falls well within this interval (Supplementary Information SI4, Supplementary Tables S1, S2). Thus, the preserved morphology makes clear the bilateral asymmetry of this axis with an underdevelopment of its right side, possibly affecting the course of the left vertebral artery (Supplementary Information SI4). Additionally, this specimen has the shortest odontoid height and a short ventral height for its superior transverse diameter within the available Neandertal sample^55^ (Supplementary Information SI4, Supplementary Figs S3, S4). This metric assessment would be consistent with a partial hypoplasia of the dens^56,57^. In modern humans, hypoplasia or even aplasia of the dens of the axis is mostly an isolated, asymptomatic defect and a possible autosomal dominant trait, but it can be associated with C1-C2 instability and neurological symptoms, and it might occur in diverse genetic disorders^57^. But since metric data fall well within the 95% prediction interval from the linear regression for the small Neandertal sample (Supplementary Fig. S4), and due to the lack of a metric reference associated with this condition in the medical literature, the presence of a hypoplastic dens remains without support.

Finally, an articulated thoracolumbar spine (SD-437) shows cranial displacement of the thoracic transitional vertebra and a sagittal cleft of the arch of the last rib-bearing vertebra with lack of development of the spinous process (Fig. 1D, Supplementary Information SI5, Supplementary Fig. S5). While cranial displacement of the thoracic transitional vertebra is common in modern humans (23%)^58^, clefting of the neural thoracic arch is rare, with few reported dry-bone cases^40,59,60^. A right rib (SD-292) is identified as either a 12th rudimentary or hypoplastic rib or a 13th lumbar rib resulting from a caudal border shifting of the thoracic-lumbar border^40,61^ (Fig. 1E, Supplementary Information SI6, Supplementary Fig. S6). Rib and vertebral anomalies may be isolated, asymptomatic findings, or may occur in association with different syndromes^62^.

### Wrist

Four of the seven scaphoids preserved at El Sidrón show morphological anomalies^63^. Three scaphoids (SDR-064, SD-258, SD-679b) retain a distinctive os centrale projection along the distoulnar border, while another scaphoid (SD-96) is bipartite with a truncated tubercle (Fig. 1F, Supplementary Information SI7, Supplementary Fig. S7). Although developmental anomalies in the human carpus are rare, these two conditions are most common^64^. Still, the occurrence of a separate or incompletely separated os centrale in modern humans ranges from 0.48% to 3.13%^65,66^, while a bipartite scaphoid is even more rare, with reports ranging from 0.13–0.60%^64,65,67–69^. The occurrence of the scaphoid anomalies in the El Sidrón is thus extraordinarily high in comparison, with 43% of seven scaphoids, or 23% of 13 individuals, presenting a distinctive os centrale portion, and 14% of seven scaphoids or 8% of 13 individuals with a bipartite scaphoid. The occurrence of os centrale and/or bipartite scaphoid in humans is often associated with congenital pathologies, including diverse syndromes like Holt-Oram, Hand-Foot-Uterus, Larsen and Oto-Palato-Digital syndromes^70–73^.

### Knee

A small, possibly left, tripartite patella (SD-932) was recovered from El Sidrón. It presents two articular surfaces inferolaterally and inferomedially for additional ossification centers (Fig. 1G, Supplementary Fig. S8). In modern humans, a bipartite patella is the most common morphological variant (frequency from 0.05% to 1.7%), while a tripartite patella is even more rare^74–76^. In both cases, the separate ossification center(s) most often occur superolaterally or laterally^74–76^, rather than inferiorly as in El Sidrón specimen. In modern humans, congenital conditions of the patella are present in more than 35 dysmorphic entities, and patella aplasia or hypoplasia is a hallmark feature of several syndromes (e.g. nail patella syndrome, small patella syndrome, isolated patella aplasia hypoplasia, Meier-Gorlin syndrome)^77^. This patella also presents other unusual morphological features that suggest a decreased or altered mechanical loading. Specifically, the SD-932 patella lacks the median patellar ridge and a well-developed subchondral bone plate, both of which are typically found in modern humans and Neandertals^78^, including other patellae from El Sidrón (Supplementary Information SI8, Supplementary Fig. S8). This patella is also distinct from the typical modern human patella and from other patellae from El Sidrón in that it presents less trabecular bone and less alignment of the struts (Supplementary Information SI8, Supplementary Fig. S8). Other non-congenital, alternative causes for these anomalous features, such as antemortem trauma^79^ or infectious processes are discarded due to absence of signs of fracture healing, bone formation secondary to trauma, or disorganized changes in the bone surface attributable to infection. Together, the morphology of SD-932 is consistent with a small, triparte patella with decreased mechanical loading, or an altered loading on at least one leg for this Neandertal individual.

### Foot

A left fully mature foot composed of *in situ* articulated bones (metatarsals 1–5, cuboid, navicular, and the three cuneiforms) and an associated talus and calcaneus were recovered at El Sidrón (Fig. 1H). The seven tarsals present a clear alteration of the plantar surface, with a general reduction of the size of the plantar half of the bones, and an organized, complementary reduction of the area of adjacent articular facets (Supplementary Information SI9, Supplementary Figs S9–S12, Supplementary Table S5). In sagittal and coronal micro-CT sections, an increase in cortical thickness is observed in the plantar surface of the cuboid, third and second cuneiform (Supplementary Figs S10,S11). The navicular, besides the complementary reduction of the articular facets for the cuneiforms, presents an abnormal shape of its tubercle and beak (Supplementary Fig. S10). Compared with other Neandertals (including El Sidrón specimens), the metatarsals also show reduced proximal articular facets for the cuneiforms and cuboid (Supplementary Figs S9,S12, Supplementary Table S5).

The described features would be less consistent with an antemortem trauma or a past episode of infection, where again signs of fracture healing, bone formation secondary to trauma, or a more disorganized and irregular reduction of the plantar border of the articular facets would be expected. The unusual shape of tarsals, the reduced articular area from the calcaneo-cuboid joint to the tarso-metatarsal joints (with continuous, well-defined and rounded plantar borders delimitating the reduced facets, that lack additional bone formation or abrupt interruptions), and the increased plantar cortical thickness of the lateral tarsals, are consistent with a congenital anomaly of the foot affecting the plantar soft tissue structures (Supplementary Information SI9) and a change of the normal load pattern of the left leg in this Neandertal. Finally, a cuboid-navicular non-osseous coalition was also observed (Fig. 1, Supplementary Information SI10, Supplementary Fig. S13).

## Discussion

**Figure 2:**
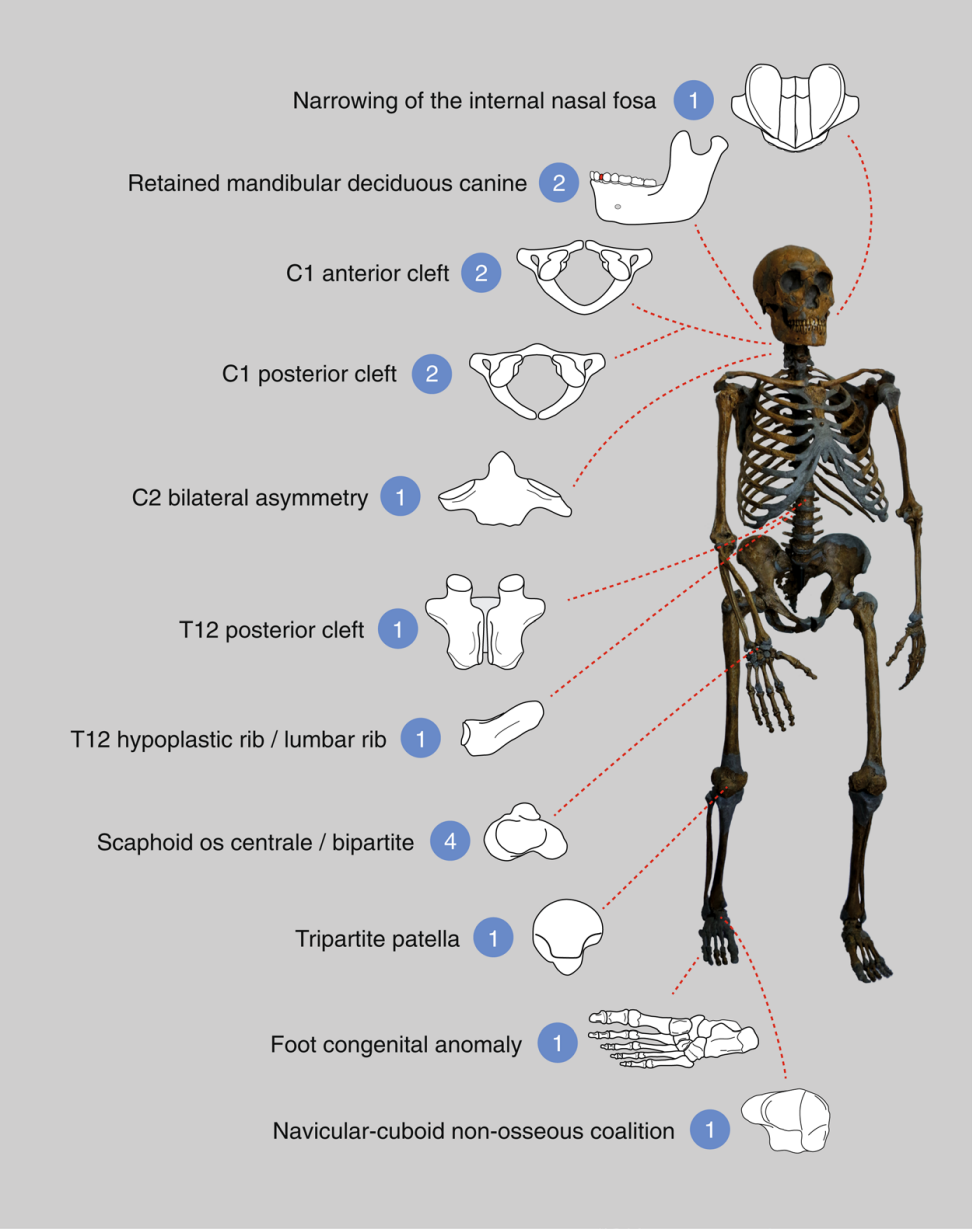
Summary of the 17 congenital anomalies observed within the El Sidrón Neandertal family group. The number of observations for each condition is shown in the blue circles, together with a schematic representation of the condition. At least four Neandertal individuals present a cleft in the arch of the first cervical vertebra.

The osteological findings presented here, together with the genetic evidence for Neandertals and specifically for El Sidrón reviewed above, constitute strong evidence for the presence of inbreeding and low biological variability in this Neandertal group. There are at least 16 congenital anomalies distributed throughout the skeleton in this group of 13 Neandertals, with at least four individuals affected by the same anomaly (Figs 1 and 2). We offer a comparison with modern human frequencies of similar conditions (Table 1) as the only available comparative data, but support the caution raised by Trinkaus^36^ in his recent, detailed review of Pleistocene hominin anomalies regarding direct comparisons between incidences in recent human and Pleistocene samples.

**Table 1:** Congenital anomalies observed within the El Sidrón family group, with frequency in modern humans and El Sidrón (percentage of individuals affected).

Anomaly	Modern humans	El Sidrón
Retained deciduous mandibular canine	0.001–1.8%^133–135^	15.38%
Nasal stenosis	0.00004%^48^	7.69%
C1 anterior cleft	0.087–0.1%^37^	15.38%
C1 posterior cleft	0.73–3.84%^37^	15.38%
C2 bilateral asymmetry	—	7.69%
T12 posterior cleft	—	7.69%
Thoracic hypoplastic rib/Lumbar rib	—	7.69%
Scaphoid os centrale	0.48–3.13%^65,66^	23.07%
Scaphoid bipartition	0.13–0.60%^64–69^	7.69%
Triparte patella	0.05–1.7%^74–76^	7.69%
Cuboid-navicular non-osseous coalition	0.2%^136^	769%
Foot congenital anomaly	—	7.69%

The health and survival consequences of inbreeding and consanguinity have been studied in humans^80–86^ and in conservation biology of endangered animal species^87–92^. In humans, it has been observed that among first cousin offspring, there is an excess of 3.5% in overall prereproductive infant mortality, with a 1.7–2.8% higher prevalence of congenital anomalies, mostly attributable to autosomal recessive disorders, several of which have been reported from communities with high consanguinity rates^84,85^. It is interesting to note that other impacts of high levels of inbreeding and consanguinity could be mediated by, for instance, an increased susceptibility to infectious diseases, with parental consanguinity as a known risk factor for primary immunodeficiencies^84,86^. No negative associations with reproductive parameters (miscarriages and fertility) have been documented, and the associations with complex diseases and quantitative traits are inconsistent^85^. Mild skeletal anomalies or variants have been observed in geographically isolated and/or endogamic human populations. For instance, in Canadian Inuit skeletons a higher frequency and intensity of several spine defects were observed in the smaller and more genetically isolated of the two compared populations^93^. In a recent study on the impact on patterns of deleterious variation of an extreme and prolonged population bottleneck in Greenlandic Inuit, an increase up to 6% in the genetic load (reduction in mean fitness in a population caused by deleterious mutations relative to a mutation-free population) was observed across all models of dominance^94^.

In a broader biological comparative context, studies on different endangered species that have suffered recent drastic population declines and range fragmentation – similar to conditions potentially experienced by Neandertals – have shown very low genetic diversity and high levels of recent, and in some cases long-term, inbreeding that can result in a reduction in population fitness, or inbreeding depression. For example, the Florida panther^88^, Scandinavian wolf^90^, and Iberian lynx^87,89^ show a range of conditions including heart defects, cryptorchidism and low semen quality^88,89,91^. The Florida panther^88^ and Scandinavian wolf^91^ also show mild dental and skeletal (mostly vertebral) anomalies that have no direct effect on fitness but are indicative of high levels of inbreeding. Mountain gorilla^92^ genetic analyses indicate a population decline over tens of millennia, recent close inbreeding and increased homozygosity, suggesting that an increased burden of deleterious mutation and low genetic diversity (including at the major histocompatibility locus, of central importance to the immune system), could have compromised the resilience of the mountain gorillas to environmental change and pathogen evolution^92^. In addition, genetic analysis of the extinct woolly mammoth reveal low heterozygosity and signs of inbreeding^95^, including several detrimental mutations^96^ in one of the last surviving mammoths. Additionally, Late Pleistocene mammoths show a high incidence of cervical ribs, a potential signal of inbreeding and/or harsh environmental conditions^97^.

In relation to the Neandertal long-term history of small and isolated populations, where the purging of deleterious alleles is predicted to be less efficient^98^, some studies have observed a larger fraction of putative deleterious alleles in Neandertals than in present-day-humans. Specifically, genes associated to autosomal recessive traits have derived homozygous genotypes with likely deleterious effects, which could be suggestive of an enrichment in recessive disorders^99^. But when both homozygous and heterozygous alleles in genes associated to autosomal recessive traits are considered, there is no clear difference between Neandertals and modern humans, and the authors conclude that the health significance of the estimated relatively (homozygous) higher genetic load in Neandertals is unclear, with no strong evidence for recessive disorders to have played a significant role in Neandertal extinction^99^. Other authors have suggested that Neandertals suffered a high load of weakly deleterious mutations, with estimations resulting in at least 40% lower fitness than modern humans on average^98,100^. With regard to the Altai Neandertal, it has been estimated that her overall genomic health was worse than 97% of present-day humans, mainly due to high risk for immune-related diseases, cancers, gastrointestinal and liver diseases, metabolic-related disorders, morphological and muscular diseases, and also neurological diseases^101^. However, these estimates of Neandertal health should not be overinterpreted since the genetic risk scores employed in the study are not deterministic^101^ and the Altai Neandertal presents greater consanguinity than that of all other Neandertal samples^31^. Furthermore, within the context of the interbreeding between Neandertals and early AMHs, the interpretation of the genomic landscape of introgression and the functional significance of Neandertal genetic material is complex and would include selection against Neandertal variants but also adaptive introgression^102–106^, including potentially adaptive ones related to the immune system^107,108^. In this regard, and in the context of the above-mentioned impact of inbreeding and consanguinity on the susceptibility to infectious diseases, it has been suggested that the transfer of pathogens between hominin populations in the Upper Paleolithic could have had negative consequences for Neandertals if these were more susceptible to some novel pathogens brought by early AMHs^109^. But although differential pathogen resistance might have played a role in the demographic collapse of the Neandertals, an explicit test of this hypothesis looking for an overall decrease in diversity at immune system loci in Neandertals failed to fully support it^110^.

Similar skeletal findings as those presented here for the Neandertals from El Sidrón are observed in some rare syndromes in modern humans, as summarized above. In several of these syndromes, the patient presents congenital anomalies in different parts of the skeleton. At El Sidrón, the maxilla, mandible, spine at different levels (C1, C2, T12), ribs, scaphoids, patella and foot are affected (Fig. 2). At least four Neandertals present congenital clefts of C1, and/or scaphoid anomalies, and it would be reasonable to expect that more than one of the congenital conditions described above could belong to the same individual (for instance the left patella and the left foot could be associated), lending some support to the presence of a syndrome. Further genetic evidence indicates a decrease of Neandertal ancestry with time in AMHs who lived between 45,000 and 7,000 years ago^111^, supporting the idea that Neandertal variants were progressively purged out. If the findings presented here are indeed related to recessive disorders or syndromes, and this scenario was not infrequent for at least late Neandertals, then this would be compatible with an initial reduction of Neandertal ancestry over time after interbreeding with early AMHs. However, although the morphological anomalies observed in the maxilla, patella and foot could have been clinically relevant, in modern humans several of the described conditions correspond to asymptomatic, incidental findings in routine medical examinations, and a diagnosis of a complex disease based on isolated skeletal elements is not possible. Thus, the possibility that the anomalies found at El Sidrón may reflect an underlying genetic syndrome remains speculative. Even in cases of consanguinity, when assessing the impact of mating between close relatives on any aspect of health, “a clear causal relationship needs to be established, rather than reliance on speculation driven solely by the presence of a close kin union in the family pedigree”^29^. Thus, whether the findings presented here constitute just a strong skeletal signal of inbreeding and low biological variability, or could be also considered as indicative of recessive disorders remains unsolved.

An alternative or compatible scenario to the interpretation of the described conditions as congenital, genetic and indicative of inbreeding would be the presence of adverse environmental conditions impacting early pregnancy and the growth period. Previous research has shown that Neandertals present nonspecific indicators of stress, such as enamel hypoplasias, at a frequency within the ranges of variation shown by prehistoric samples of modern human foragers^112^. Specific evidence from El Sidrón^113^ indicates that the inspection of all teeth resulted in all dental individuals presenting enamel hypoplasia (incisors 59%, canines 50%, premolars 58%, and molars 32%), although with varying degrees of intensity and within the frequencies observed in modern human historical samples for the incisors and canines^114,115^. Furthermore, previous analysis of the El Sidrón J1 juvenile skeleton indicated that the dental and skeletal growth and maturation values were similar to those of diverse modern human juvenile populations^14^, while the size and shape studies of the adult postcranial remains from El Sidrón show that these Neandertals fall well within the range of variation documented for this Paleolithic humans^116–119^. Together, these analyses offer limited support for unusually harsh environmental conditions impacting the prenatal and/or postnatal growth period as an explanation of the described anomalies in the Neandertals from El Sidrón. This is consistent with Trinkaus’ recent assessment of developmental anomalies and abnormalities in the Pleistocene hominin fossil record, in which he concludes that stress during development could only account for a few of the observed abnormalities^36^.

Current examples of animal species with a long-term history of low population size and depleted genetic diversity may indicate a resilience to develop strategies to mitigate the effect of inbreeding^87,92^. Therefore, caution has been advised when drawing conclusions about the reasons for Neandertal extinction^98^, since Neandertals could have evolved diverse genetic and biocultural compensations to cope with a large deleterious genetic load^110^. The persistence of Neandertals for tens of thousands of years, with increasing evidence for diverse subsistence strategies^6,7^, symbolic behavior and complex technologies^8–13^ and healthcare^120^ (several of this examples coming from El Sidrón), demonstrates the resilience of these Paleolithic hominins.

The Adult 2 Neandertal from El Sidrón exemplifies this resilience. This individual had a congenital narrowing of the nasal fossa and a retained deciduous mandibular canine with a subsequent dentigerous cyst secondary to dental trauma to that tooth. Previous analysis of the dental striation orientation^99^ suggest that this individual coped with their dental pathology by alternating left-right hand use and avoiding chewing on the pathological side of the mouth^121^. Adult 2 also presented the highest incidence of chipping in the dentition, and was the only individual from El Sidrón from whom bitumen or oil shale was recovered from the dental calculus^122^. Together, these findings suggest that this Neandertal individual could have had a specialized behavior. Furthermore, previous studies have found evidence that this individual probably self-medicated the infected cyst^7,122^. Considering the El Sidrón Neandertal group as a whole, and the findings presented here from the perspective of the bioarchaeology of care in Neandertals^120^, any interpretation of these findings as related to a genetic syndrome or recessive disorder could point to another example of healthcare and resilience in this hominin group.

A scenario of small effective population sizes, a hunter-gatherer existence and population dispersal into separate small kindred groups, with probable intragroup mating, would have also affected early AMHs. Even considering the interbreeding episodes with early AMHs, and therefore somehow the permanence of Neandertals, how early AMHs managed to support growing and geographically-expanding populations in the same environment while the Neandertal phenotype disappeared, remains a central question. The genetic analysis of a 45,000 years old modern human male from Siberia indicates lack of recent inbreeding among his ancestors^123^, and the genomes analyzed from the Sunghir site, dated 34,000 years ago, indicate that they were not closely related (third degree or closer)^124^. Beyond the osteological and paleopathological analysis of the skeletons from Sunghir that could point to inbreeding^125^, the genetic results of these individuals have been interpreted as already suggesting by 34,000 years ago the presence of the modern human hunter-gatherer social structure “with low levels of within-band relatedness, complex family residence patterns, relatively high individual mobility, and multilevel social networks”^124^. Currently, the available osteological evidence indicates absence of differences of younger versus adult mortality pattern between Neandertals and early AMHs^25^, in agreement with biological models that would indicate similar demographic features between both^126^, and this would suggest that the demographic advantage of early AMHs would have been the result of increased fertility and/or reduced immature mortality^25^. Fertility and immature mortality would be related to the social structure of Paleolithic hominins, and the study of the impact of a reduced population dispersed in small an isolated groups on that social structure, as well as the study of the reproductive biology of hominins remains an important challenge for the future^127^.

The disappearance of the Neandertals and expansion of modern humans was most probably the result of a process involving several factors, one of them being the low population density of Neandertals. An analysis of the climatic niches of both Neandertals and early AMHs^128^ indicates that from 48 to 40 ka, the potential niche of Neandertals reduced significantly in size and spatial continuity (connection between optimal habitat patches), while optimal patches of early AMHs remained much better connected. Neandertal habitat reduction and fragmentation suggest that the Neandertal population was sharply decreasing in size and becoming more isolated^128^, a conclusion that is generally supported by paleogenetic data. The analysis of the genomes of Neandertals (Vindija33.19 and Altai), Denisovans and modern humans by Pairwise Sequential Markovian Coalescent (PSMC) method, indicates that after a reduction of population size that occurred sometime before 1.0 million years ago, the population ancestral to present-day humans increased in size, whereas the demographic history for both Neandertals and the Denisovan shared a recent history of very low effective population sizes^31,129^. Recent estimations suggest that in five low-coverage Neandertal genomes who lived around 39,000 to 47,000 years ago (Les Cottés, Goyet Q56-1, Mezmaiskaya 2, Vindija 87, Spy 94a)^130^, the levels of heterozygosity lie below those estimated from the high-coverage Vindija and Altai Neandertals, from around 50,000 years ago or at least predating 44,000 years (Vindija)^131^. The possibility of particularly low heterozygosity in these late Neandertals could reflect a small number of individuals near the end of its presence in Europe^132^. At 49,000 years ago, and in the context of a high incidence of skeletal anomalies in Pleistocene *Homo*^36^, the Neandertal family group from El Sidrón, with genetic and skeletal evidence of inbreeding, could be representative of the beginning of the demographic collapse of this hominin phenotype.

## Material and Methods

The bones were inspected using a binocular lens, a Enviromental Scanning Electron Microscope (ESEM Fei-Quanta 200), and were micro-CT scanned with a Nikon XT H 160 at 155-114 kv and 48–85 µA, 1800 projections, reconstructed as 16-bit tiff stacks, voxel size interval from 0.027 to 0.079 mm. The data were loaded into AMIRA 5.4® (Thermo Fisher Scientific) for generating the virtual reconstructions. Photographs were obtained from different views and, when possible, with comparative Neandertal (El Sidrón) and modern human cases. Descriptions of specific procedures followed for the morphological analyses for each skeletal element are presented in the Supplementary Material.

## Supplementary information

SUPPLEMENTARY_INFORMATION

## Data Availability

All data generated or analysed during this study are included in this published article (and its Supplementary Information files).
