# Supplementary material for: Skeletal Anomalies in The Neandertal Family of El Sidrón (Spain) Support A Role of Inbreeding in Neandertal Extinction
Source: Sci Rep. 2019 Feb 8;9:1697. doi: 10.1038/s41598-019-38571-1 (PMC6368597; doi:10.1038/s41598-019-38571-1)
Supplement: Supplementary file 1 — SUPPLEMENTARY_INFORMATION [file 41598_2019_38571_MOESM1_ESM.doc]

**SUPPLEMENTARY INFORMATION FOR**

SKELETAL ANOMALIES IN THE NEANDERTAL FAMILY OF EL SIDRÓN (SPAIN) SUPPORT A ROLE OF INBREEDING IN NEANDERTAL EXTINCTION

Ríos L^a,*^, Kivell TL^b,c^, Lalueza-Fox C^d^, Estalrrich A^e^, García-Tabernero A^f^, Huguet ^g,h^, QuintinoY^i^, de la Rasilla M^j^, Rosas A^f,*^

^a^Department of Physical Anthropology, Aranzadi Zientzia Elkartea, Zorroagagaina 11,

20014 Donostia, Gipuzkoa, Basque Country, Spain; ^b^Skeletal Biology Research Centre, School of Anthropology and Conservation, University of Kent, Marlowe Building, Canterbury, UK CT2 7NR; ^c^Department of Human Evolution, Max Planck Institute for Evolutionary Anthropology, Deutscher Platz 6, Leipzig, Germany 04103; ^d^Institute of Evolutionary Biology (CSIC–Universitat Pompeu Fabra), Carrer Dr. Aiguader 88, 08003 Barcelona, Spain; ^e^Instituto Internacional de InvestigacionesPrehistóricas de Cantabria IIIPC (Universidad de Cantabria, Santander, Gobierno de Cantabria). Avda. de los Castros 52, 39005 Santander, Cantabria, Spain; ^f^Paleoanthropology Group, Department of Paleobiology, Museo Nacional de Ciencias Naturales (MNCN-CSIC). José Gutiérrez Abascal 2, 28006 Madrid, Spain; ^g^IPHES, Institut Catala de Paleoecologia Humana i Evolució Social, Campus Sescelades URV (Edifici W3), 43007 Tarragona, Spain; ^h^Area de Prehistoria, Universitat Rovira i Virgili, Avda. Catalunya 35, 43002 Tarragona, Spain; ^i^Unidad asociada al CSIC, Departamento de Paleobiología, Museo Nacional de Ciencias Naturales, Calle José Gutierrez Abascal 2, 28006 Madrid, Spain; ^j^Laboratorio de Evolución Humana, Dpto. de Ciencias Históricas y Geografía, Universidad de Burgos, Edificio I+D+i, Plaza Misael Bañuelos s/n, 09001 Burgos, Spain; ^k^Área de Prehistoria Departamento de Historia, Universidad de Oviedo, Calle Teniente Alfonso Martínez s/n, 33011 Oviedo, Spain.

^*^To whom correspondence may be addressed:

Luis Ríos, Department of Physical Anthropology, Aranzadi Zientzia Elkartea, Zorroagagaina, 11, 20014 Donostia, Gipuzkoa, Basque Country, Spain. Email: [mertibea@yahoo.com](mailto:lrios@mncn.csic.es)

Antonio Rosas, Paleoanthropology Group, Department of Paleobiology, Museo Nacional de Ciencias Naturales (MNCN-CSIC). José Gutiérrez Abascal 2, 28006 Madrid, Spain. Email: arosas@mncn.csic.es

**This PDF file includes:**

Supplementary Information SI1 to SI10

Supplementary Figs. S1 to S13

Supplementary Tables S1 to S5

**SI1. CONGENITAL ANOMALIES**

In the paleopathological literature[[1]](#footnote-2), an anomaly is defined as “a non-pathological deviation from normal structure”. This definition would apply to anatomical variants classically termed as discrete, non-metric or epigenetic traits, although the limit between pathological and non-pathological could be not so well defined for some of these classic traits^1-3^. Following Barnes (2012)^4^, we expand the definition of anomaly to include both pathological and non-pathological bone variants deviating from designated standard ranges of variation.

Our main objective was not to obtain a differential diagnosis of the observed anomalies (for instance see below for the maxilla, second cervical vertebra and left foot), but to describe and identify the anomaly as congenital and genetic. Congenital has been defined as “a condition that is manifest at birth or if manifest postnatally, is considered to have been present at birth and attributable to events in utero” (Nomenclature in Paleopathology ^*^), while genetic has been defined as “pertaining to an inherited condition” (Nomenclature in Paleopathology ^*^). Here, the combined use of both definitions indicates that the anomaly was manifest at birth or postnatally and in both cases was mostly attributable to a genetic cause. In some cases the identification of the anomaly did not imply a differential diagnosis but a correct description (for instance see below for the os centrale, the thoracic hypoplastic or lumbar rib and the non-osseous calcaneo-cuboid coalition). For the association of the observed anomalies with a specific condition or group of conditions, we used the expression “consistent with”, defined as follows “the lesion could have been caused by the condition(s) described, but it is non-specific and there are many other possible causes”^5^. We chose this definition since no clear associations between the presented bones could be concluded, and the assessment of isolated bones generally precludes the use of terms as “highly consistent”, “typical of” and “diagnostic of”^5^, indicative of an increasing degree of certainty of the diagnosis. Furthermore, we would like to emphasize that the definition of “consistent with” includes that the lesion “is non-specific and there are many other possible causes”. Within these other possible causes we address, as we did previously^6^, traumatic pathology, infectious conditions, adverse environmental conditions impacting early pregnancy and the postnatal growth period (see main text), and taphonomic alterations.

With regard to the latter, an important step in the description of the anomalies was to discard taphonomic processes as a cause of the alterations. Mechanic or chemical postmortem alterations of the bones would be characterized by a disorganized, irregular or abrupt changes of the outer surfaces of the bone, with exposition of cortical discontinuities and trabeculae (especially in the case of mechanical alterations). Binocular lens, SEM and micro-CT were used to discard this kind of modifications, and we proceeded with caution, drawing non-conclusive observations when it was not possible to fully discard taphonomic processes as important for the observed bone anomalies.

**SI2. MAXILLA AND MANDIBLE**

The Internal Nasal Fossa Breadth (INFB), defined as "the greatest distance between the lateral nasal walls of the nasal cavity at the level of the inferior nasal turbinate"^7^, was recorded in the three preserved maxillae from El Sidrón, including Adult 2. The measurement was taken in the middle portion of the internal nasal fossa for El Sidrón A2, and Adult 3 and Adult 5 (A3 and A5) (Supplementary Table S1). A mean INFB of 34.5 mm (SD=4) for 7 Neandertal maxillae (Arcy-sur-Cure 9, La Chapelle-aux-Saints 1, Gibraltar 1, Guattari 1, Krapina 47, Vindija 225, Vindija 259) has been previously published ^8^. Considering El Sidrón A3 and A5 as normal maxillae, their INFB values (31.2 and 34.3 mm) fall within the mean ± 1SD Neandertal values (30.5-38.5 mm). Adding both cases to the previous Neandertal sample^8^, the total Neandertal mean (n=9) would be 34.12 mm (SD=3.6). In comparison with both means, El Sidrón A2 at 22.47 mm falls at the smallest extreme of the observed interval of variation of Neandertal INFB (Supplementary Table S1). EL Sidrón A2 also falls at the smallest extreme of variation in modern human populations^7^, with a mean of 32.87 mm for the complete sample, including at the extreme of the Arctic population, which has the smallest INFB among modern humans with a mean of 30.3 mm (Supplementary Table S1). The reduced INFB of El Sidrón A2 (Supplementary Fig. S1A) is consistent with a congenital narrowing of the nasal fossa in this Neandertal.

The two other anomalies described for this maxilla are the right-side deviation of the anterior nasal crest of the maxilla along its entire length (Supplementary Fig. S1A-B), and the asymmetry of the dental arcade (Supplementary Fig. S1A). Interpretation of the deviation of the anterior part of the crest is complicated by the presence of fractures affecting the anterior part of the maxilla. These fractures caused the posterior displacement of the outer cortex of the maxilla corresponding to the alveoli of both central incisors and the left lateral incisor. The fact that the roots of these three teeth are not affected by any fracture would indicate that these fractures of the maxillary cortex occurred postmortem, after the three teeth fell out of their alveoli. Thus, it seems that at least part of the alterations observed around the nasal aperture corresponds to postmortem fractures. The right-side deviation of the anterior part of the maxilla’s anterior nasal crest does not appear to be the result of perimortem trauma due to the lack of any visible bone fracture or discontinuity affecting the crest properly (Supplementary Fig. S1A-B). Antemortem trauma cannot be ruled out, although we would expect further alterations affecting the outer cortex of the maxilla from an applied force able to produce such deviation at the nasal aperture. With regard to the posterior part of the crest, the right-side deviation initiates at its posterior limit (Supplementary Fig. S1A-B), with no clear evidence of bone remodeling or perimortem or antemortem fracture. The bilateral asymmetry of the dental arcade (Supplementary Fig. S1A) does not seem to be caused by postmortem alteration, but we caution that a definitive interpretation of both features as congenital anomalies is difficult due to potential taphonomic processes.

The narrowing of the nasal fossa is consistent with a congenital condition, and could be consistent with several specific conditions like congenital nasal pyriform aperture stenosis^9-11^, choanal atresia^12^, and Goldenhar^13^, Aper^14^ and Binder^15^ syndromes. However, most of these conditions present with anomalies in other regions of the craniofacial and postcranial skeleton. Since no clear anomalies are observed in the preserved teeth, and due to the above mentioned potential taphonomic alterations to the maxilla, we would consider that complex syndromes like Goldenhar, Aper and Binder, would be unlikely based on current evidence.

| **Individual / Samples** | **INFB** | **INFB** |
| --- | --- | --- |
| Arcy-sur-Cure 9 | 35 | 35 |
| La Chapelle-aux-Saints 1 | 34 | 34 |
| Gibraltar 1 | 35 | 35 |
| Guattari 1 | 39 | 39 |
| Krapina 47 | 39.6 | 39.6 |
| Vindija 225 | 30 | 30 |
| Vindija 259 | 29 | 29 |
| El Sidrón Adult 1 | - | 31.2 |
| El Sidrón Adult 2 | - | 34.3 |
| Neandertal mean, SD | 34.51, 4 | 34.12, 3.6 |
| Modern human mean | 32.86 | |
| Arctic population mean, SD | 30.3, 2.5 | |
| El Sidrón Adult 2 | 22.47 | |

**Supplementary Table S1**. Neandertal and modern human individual and mean values for INFB, compared with values from El Sidrón A2, A3 and A5.

**Supplementary Fig. S1**. The maxilla of El Sidrón Adult 2. A: from top to bottom, superior, frontal and inferior view of the maxilla. In the superior view, the reduced size of the nasal fossa can be observed. In both the superior and frontal views, the right-side deviation of the anterior nasal crest of the maxilla along its entire length can be observed. In the inferior view, the asymmetry of the dental arcade is visible. B: two coronal micro-CT sections of the maxilla at the levels indicated by the blue arrows in A, showing the right-side deviation of the crest of the maxilla and the continuity of bone tissue between the anterior nasal crest and the nasal fossa floor.

**SI3. FIRST CERVICAL VERTEBRA**

SD-636 is a portion of the right anterolateral quadrant of a first cervical vertebra (C1) that preserves the anterior half of the superior and inferior articular surfaces, as well as the right half of the facet for the dens of the axis (Supplementary Fig. S2A). This fragment presents a congenital anterior sagittal cleft of C1. The facet for the dens of the axis was divided by this cleft, and the right half of the facet is clearly defined by a vertical straight line (i.e. it is not broken) (Supplementary Fig. S2B-D). Medial to this half-facet, ventral-to-dorsal bone continuity can be observed through the sagittal plane (Supplementary Fig. S2E-G). In total, four atlases out of five with observable anterior or posterior sagittal arches (80% of the atlases from El Sidrón) present congenital clefts of the atlas, as shown in Supplementary Fig. S2A.

**Supplementary Fig. S2**. Four first cervical vertebrae from El Sidrón with congenital clefts. A: From left to right, a superior view of the two C1 hemi-arches from El Sidrón J1 (SD-2045 and SD-1725, posterior clefts), and the posterior and superior views of SD-1643 (posterior cleft), SD-1094 and SD-636 (anterior clefts). B-D: From left to right, posterior view of the facet for the dens of the axis of SD-636, with SEM images from the locations indicated by the red asterisks, where the straight, vertical delimitation of the facet can be observed. E-G: From left to right, medial view of the sagittal cleft of SD-636, with SEM images from the locations indicated by the red asterisks, where continuity of the cortical bone can be observed in the sagittal plane.

**SI4. AXIS C2**

The sagittal and transverse diameters of the transverse foramen (TF) of SD-1601 were compared with modern human data to quantify its bilateral asymmetry (Supplementary Fig. S3A-B). The metric values are presented in Supplementary Table S2 demonstrating that the values for the right TF of SD-1601 are at the smallest extreme of variation observed in three modern human samples, while values for the left TF fall well within the modern human interval.

| **Measurement** | | **Taitz^16^**  **N=33** | | **Madawi^17^**  **N=50** | **MNCN**  **N=23** | | **SD-1601** | |
| --- | --- | --- | --- | --- | --- | --- | --- | --- |
| **TF** | **Lateral** | L | R | L+R | L | R | L | R |
|  | **Sagittal** | 5.76, 0.71  (4-7.3) | 5.85, 1.39  (3.9-11.3) | 5.7, 1  (3.9-9) | 5.75, 1  (4.14-7.9) | 5.65,0.68  (4.07-7.05) | 4.5 | 3.5 |
|  | **Transverse** | 4.99, 0.66  (3.4-6) | 4.77, 0.7  (3.6-6.2) | 5.6, 0.9  (4.3-8) | 4.83, 0.74  (3.49-6.15) | 4.99, 0.78  (3.87-7) | 5.5 | 3.6 |
|  | **Inferior** | L | R |  | L | R | L | R |
|  | **Sagittal** | 7.5, 1.17  (4.7-10.8) | 6.75, 0.84  (4.5-8.2) |  | 6.16, 0.78  (4.83-7.76) | 5.84, 0.88  (4.57-7.82) | 7.77 | 2.98 |
|  | **Transverse** | 5.71, 0.59  (4.4-6.7) | 5.26, 0.76  (3.4-6.6) |  | 6.76, 1.29  (4.68-9.56) | 6.47, 1.12  (4.42-8.71) | 5.71 | 2 |
| Lamina | | | | | 6.02, 1.19  (4.19-8.65) | 5.75, 1.21  (3.96-8.43) | 6.55 | 3.9 |

**Supplementary Table S2**. Diameters of the TF and thickness of the lamina of the axis in three modern human samples and the Neandertal vertebra SD-1601. Measurements in mm. Mean, SD (minimum-maximum values). L=left, R=right; MNCN= modern human sample from the Museo Nacional de CienciasNaturales (Madrid, Spain)

In modern humans there is some asymmetry in TF size within the cervical vertebrae, with the left TF usually being larger than the right^16-18^. However, this difference in size only reaches statistical significance in some studies for the C3-C6 segment^18^ and rarely in C2^17^. In the modern human sample measured here (MNCN), the bilateral asymmetry between the dimensions of the TF was evaluated. First, the area of each foramen (right/left, lateral/inferior) was calculated following the formula for estimation of the area of an ellipse (*A=πab*, a and b the semi-minor and semi-major axes of the ellipse or diameters of the TF). Then, statistical tests were applied (related-samples Wilcoxon signed-rank test, paired samples t-test), to the linear measurements and the areas. No significant bilateral asymmetry was found. The mean values can hide patent asymmetrical cases, so asymmetry was calculated for the areas of the TF following two formulae, one for relative asymmetry^19^ and other for absolute asymmetry[[2]](#footnote-3). Results are shown in Supplementary Table S3. The asymmetry values for the areas of the TF of the SD-1601 Neandertal axis fall outside the interval observed in this modern human sample. This observation should be interpreted with caution due to the small size of the modern human sample.

|  | **Absolute asymmetry** |  | **Relative asymmetry** |  |
| --- | --- | --- | --- | --- |
|  | **MNCN n=23** | **SD-1601** | **MNCN n=23** | **SD-1601** |
| **Lateral TF** | 5.06, 55.11  (-79.69 / 59.97) | -96.43 | 49.36,483.01  (-569.87 / 461.35) | -650.6 |
| **Inferior TF** | -1.41, 44.79  (-136.95 / 57.77) | -644.41 | -14.01, 400.94  (-812.87 / 448.24) | -1526.29 |
| **Lamina** | -3.33, 22.63  (-67.65/ 50) | -67.95 | -25.76, 176.27  (-377.51 / 154.67) | -507.18 |

**Supplementary Table S3**. Observed asymmetry values for the areas of the lateral and inferior TF, and the thickness of the lamina in a modern human sample (MNCN) and SD-1601. Median, interquartile range, (minimum-maximum values).

The bilateral asymmetry of the vertebra can be also observed in the hypoplasia of the right transverse process (TP) (Supplementary Fig. S3A). The right TP can be seen in its normal location, with a rounded shape similar to the left TP, but reduced in volume. This asymmetry extends to the posterior region of the axis, where a clear asymmetry of the thickness of the lamina can be observed (Supplementary Fig. S3A-C). The value of the thickness of the right lamina falls at the extreme of modern human variation, while the thickness of the left lamina falls well within this interval (Supplementary Table S2). The asymmetry values (Supplementary Table S3) fall at the extreme (absolute asymmetry) or beyond the interval observed in the modern human sample (relative asymmetry). Again, this observation should be interpreted with caution due to the small size of the comparative sample. Finally, absence of development of the right half of the tip of the spinous process can be also observed (Supplementary Fig. S3A,B,D).

A clear bilateral asymmetry can be observed in this Neandertal vertebra. On the basis of the asymmetry observed in the TF, it is possible that the vertebral artery (VA) was reduced in size, although the occupancy ratio between the VA and the TF is variable^20,21^. If this was the case, smaller VAs are associated with anomalous aortic origin or an abnormal entrance of the VA on the TF^22^. Alternatively, or additionally, the VA could have been fenestrated^23^. In this case, the small right TF could represent the course for the smallest channel of the artery, while the larger channel could have been canalized outside the TF, similar to some cases of first intersegmental artery^24^. However, no cases of VA anatomical variants at the axis TF in the form of fenestration or duplication have been found in the literature.

The tip of the odontoid process presents an irregular form. Analysis of this surface with binocular lens, as well as micro-CT imaging of sagittal and coronal sections (Supplementary Fig. S3E), rule out postmortem modifications as the cause of the irregular form. No trabeculae were exposed, no abrupt discontinuities or irregular bone destruction are observed, and the surface is smooth. The odontoid process is also short. A metric comparison of the dimensions of SD-1601, using previous measurements and valueson hominin cervical vertebrae^25,26^, demonstrates that SD-1601 presents a short ventral height both in absolute terms (the third shortest among eight Neandertals), and in comparative terms in relation with its superior transverse diameter. It is the shortest axis for its transverse diameter, although well within the 95% prediction interval from the linear regression for the small Neandertal sample (Supplementary Fig. S4A, n=7). SD-1601 also presents the shortest odontoid height relative to the height of the vertebral body within the Neandertal sample, and since the inferior epiphyseal ring is unfused, its odontoid height is a slight underestimation. In comparison with body height, odontoid height falls well within the 95% prediction interval from the linear regression for the small Neandertal sample (n=7) (Supplementary Fig. S4B).

The short height of the axis and its odontoid process could be related to a possible hypoplastic dens, but due to the metric differences in the axis between modern humans and Neandertals and the lack of a clear metric reference associated with this condition in the clinical literature, the presence of an hypoplastic dens remains without support^27^.

In summary, the observations on the transverse foramina, transverse processes, laminae and tip of the spinous process of SD-1601 are highly consistent with a congenital bilateral asymmetry of this vertebra towards an underdevelopment of its right side, possibly affecting the course of the left vertebral artery.

**Supplementary Fig. S3**. A: Inferior view of the axis, where the bilateral asymmetry of the transverse process (blue triangles), transverse foramen (TF), lamina and spinous process can be observed. B: axial micro-CT section of the axis where the course of both TF (blue arrows), and their different diameters, as well as the asymmetry of the laminae can be observed. C: micro-CT section of both laminae at the level indicated by the blue arrows in A. The difference in the thickness of the lamina diameters (subperiosteal and endosteal) can be observed. D: sagittal micro-CT sections of the right (absent) and left halves of the tip of the spinous process at the level indicated by the blue arrows in A. Cortical bone on the posterior surface can be observed (blue triangles) . E: anterior view of the axis, and coronal and sagittal micro-CT sections where the short odontoid process and body can be observed.

**Supplementary Fig. S4**. Metric comparison of SD-1601 with other Neandertals and modern humans. A: Ventral height and superior transverse diameter. B: Odontoid height and body height. Linear regression lines and 95% prediction intervals are shown.SD-1601 is indicated by the green arrows.

**SI5. THORACIC VERTEBRA**

The last rib-bearing vertebra in SD-437, an articulated portion of the thoracolumbar spine, shows a posterior cleft and the lack of development of the spinous process. Inspection of the morphology through a binocular lens and CT imaging were performed to assess the likelihood of postmortem fracturing and/or erosion as the cause of the cleft observed in the posterior arch. Continuity of cortical bone was observed in both laminae through binocular lens. This observation was supported by CT-scan sections at different levels of the laminae (Supplementary Fig. S5C-D), where the cortical bone was observed along the perimeter of the cleft in both laminae, suggesting the abnormal morphology was congenital. In addition to the congenital cleft, this vertebra lacked the development of the spinous process (Supplementary Fig. S5B).

**Supplementary Fig. S5**. Posterior (A) and lateral (B) views of the last rib-bearing vertebra of SD-437. The posterior cleft and the lack of development of the spinous process can be observed. In the lateral view, the lumbar pattern of the superior and inferior articular facets can be observed, together with the mamillary process (blue triangle) and the costal facet (green triangle) (B). Axial sections of the laminae at two levels (C and D) show the continuity of the cortical bone in both laminae around the cleft (small blue triangles).

**SI6. THORACIC HYPOPLASTIC OR LUMBAR RIB**

The right rib SD-292, identified either as a 12th rudimentary or hypoplastic rib or as a 13th lumbar rib is shown in posterior, anterior, posterior, superior and inferior views in Supplementary Fig. S6.

**Supplementary Fig. S6**. Posterior (upper left), anterior (upper right), inferior (bottom left) and superior (bottom right) views of SD-292

**SI7. WRIST**

Three of the seven scaphoids found at El Sidrón retain a distinctive os centrale projection along the distoulnar border (SDR-064, SD-258, SD-679b). In modern humans, a separate os centrale can occur in three ways: (1) as a well-developed, independent bone; (2) present radiographically as an empty space between the capitate and trapezoid; or (3) as an incompletely separated bony fragment of the scaphoid^28^. The morphology of the three El Sidrón scaphoids is most similar to the third condition (Supplementary Fig. S7A,B). A distinctive os centrale portion is also found in other Neandertal scaphoids^29,30^ but has not been formally discussed to our knowledge. One scaphoid (SD-96) is bipartite with a truncated tubercle. Bipartite scaphoids can be the result of trauma^31^ (i.e. fracture). Several criteria have been defined in the clinical literature to differentiate a congenital bipartition from a fracture (bilaterality, no history of wrist injury, clear-cut gently rounded edges of the bones, equal density of both parts, no evidence of degenerative changes, no signs of bone marrow enema and visualization of cartilage), and some cases of congenital bipartite scaphoid have been identified^32-35^. SD-96 presents an articular facet with a regular contour, rounded edges with no degenerative changes, and a smooth joint surface (Supplementary Fig. S7C), whereas in contrast, the articular surfaces resultant from trauma present a more irregular contours and contact surfaces (Supplementary Fig. S7D). Thus, the morphology of SD-96 is consistent with a congenital bipartite scaphoid condition.

**Supplementary Fig. S7. A**: The distinctive os centrale portion (at top of image) in three scaphoids from El Sidrón (top to bottom, SD-258, SDR-064, SD-679b), medial (left) and radial (right) views. B: a right scaphoid from an archaeological case with a “partly marked off” os centrale in medial view. C: SD-96, the bipartite scaphoid, in radial (top), distal (middle) and palmar (bottom) views. D: two archaeological cases with unilateral fracture of the scaphoid, before and after the reconstruction.

**SI8. KNEE**

The articular breadth (maximum mediolateral diameter of the facet) of the tripartite patella SD-932(28.69 mm) places it at the smallest extreme of the Neandertal range of variation (mean 43.70, SD= 4.18)^36^, with a maximum mediolateral diameter of the patellar body only larger than Tabun 1, a female Neandertal (Supplementary Table S4). The two facets for the additional ossification centers present rounded edges and some pitting in the inner area (Supplementary Fig. S8A), similar to non-osseous coalition in the foot bones (see section 9).

The lack of a median patellar ridge separating the lateral and medial articular facets can be observed in the transverse micro-CT section of SD-932 (Supplementary Fig. S8D), while lack of a well-developed subchondral bone plate can be observed in the sagittal and transverse micro-CT sections (Supplementary Fig. S8B,D). Both of these features are well-developed in the other morphological normal patellae from El Sidrón (see Supplementary Fig. S8B-D for specimen SD-1718 as an example), and is typical of patellae subjected to normal loading patterns^37^. The sagittal, coronal and transverse micro-CT sections also reveal, although there are inclusions within the internal bone structure, a limited amount of trabecular bone within SD-932 and less alignment of the trabecular struts (isotropic) than that of the comparative SD-1718 (Supplementary Fig. S8B-D).

| **Case** | **Articular breadth** |
| --- | --- |
| Krapina 215.1 | 49.5 |
| Krapina 215.2 | 44 |
| Krapina 215.3 | 44 |
| Krapina 215.5 | 41 |
| Krapina 216.1 | 48 |
| Krapina 216.3 | 43 |
| Krapina 216.4 | 47 |
| Krapina 216.6 | 41 |
| Krapina 216.8 | 45 |
| Krapina 216.9 | 43 |
| La Chapelle 1 | 42.5 |
| La Ferrassie 2 | 39 |
| Kiik-Koba 1 | 48.5 |
| Régourdou 1 | 43 |
| St Césaire 1 | 46.5 |
| Shanidar 1 | 47.5 |
| Shanidar 4 | 47 |
| Shanidar 5 | 45 |
| Spy 2 | 50.5 |
| Subalyuk 1 | 43.5 |
| Tabun 1 | 33 |
| SD-1718 | 39.96 |
| SDR-098 | 44.39 |
| SD-630 | 42.15 |
| SD-1226a | 34.71 |
| Neandertal mean, SD | 43.7, 4.18 |
| SD-932 | 28.69 |
| SD-932t | 34.19 |

**Supplementary Table S4**. Articular breadth (maximum mediolateral diameter of the facet) values from Neandertals^36^ and from patellae from El Sidrón. Measurements for SD-932 include the articular breadth and the maximum mediolateral diameter (SD-932t), since in the lateral and medial borders of the patella extend well beyond the articular facet.

**Supplementary Fig. S8**. Anterior, posterior, medial and lateral views of SD-932 (A). Micro-CT sagittal, coronal and transverse sections of SD-932 and SD-1718, another patella from El Sidrón that appears morphologically normal (B,C,D).

**SI9. FOOT**

The abnormal anatomy of each bone from the left foot is comparatively described according to standard anatomical description and nomenclature of the bones of the foot^38,39^.

**Metatarsals**

Metatarsals 2-5 show a reduction in the area of the proximal articular facet. A metric comparison with other Neandertal metatarsals was undertaken, focusing on the fourth metatarsal metrics due to the availability of data from the literature^40,41^. Metric data from other fourth metatarsals from El Sidrón were included (Supplementary Table S5). For its total length, the proximal articular breadth of the fourth metatarsal falls well within the 95% prediction interval from the linear regression for the Neandertal sample, while it presents a clear reduction in its proximal articular height, which places it outside the 95% prediction interval (Supplementary Fig. S9). These results are in agreement with the general reduction in bone size observed in the plantar aspect of the bones from this foot.

**Cuboid**

The plantar surface of the morphologically typical cuboid presents a strong ridge, the tuberosity of the cuboid, which divides this surface into a small anterior and flat area, and a large posterior area^38^. The plantar surface provides attachment to several ligaments (plantar cubometatarsal_5_, cubometatarsal_4_, longitudinal plantar ligament, short plantar calcaneocuboid, plantar cubonavicular, and plantar cuneo_3_-cuboid), as well as to the peroneus longus fibrous tendon sheath, an expansion from the tibialis posterior tendon, opponens and short flexor of the fifth toe, the oblique head of the adductor hallucis, and the flexor hallucis brevis^38^. As indicated elsewhere^38^, some of these latter attachments are related to the fibrous tunnel of the peroneus longus tendon. The cuboid presents articular facets for the fourth and fifth metatarsals (anterior surface), the calcaneum (posterior surface), and the third cuneiform and navicular (medial surface). The normal morphology of the tuberosity and articular facets can be observed in a comparative cuboid from El Sidrón, SD-1033 (Supplementary Fig. S10A).

| **Individual** | **Total length (M2)** | **Proximal articular breadth (M6b)** | **Proximal articular height (M7b)** |
| --- | --- | --- | --- |
| Kebara_9 | 60.6 | 7.1 | 13.3 |
| Ferrassie_1 | 75.6 | 14 | 18.1 |
| Ferrassie_2 | 63 | 12.3 | 18.5 |
| Ferrassie_2 | 62.1 | 10.7 | - |
| Regourdou | 73.4 | 12.5 | 20.4 |
| Krapina 248.3 | - | 11 | - |
| Krapina 248.2 | 73.6 | - | - |
| Krapina 248.1 | 71.5 | - | 16 |
| Suba_yuk_1 | 66 | 10.4 | 16 |
| Kiik_Koba | 72.6 | 14.8 | 22.3 |
| Kiik_Koba | 72.9 | 15.4 | 22.3 |
| Shanidar_1 | 71 | 11 | 19 |
| Shanidar_6 | 65.4 | - | - |
| Shanidar_8 | 64 | - | - |
| Tabun | 65.2 | 11.4 | - |
| SD-1048 | - | 12.96 | 19.72 |
| SD-1043 | 76.2 | 11.48 | 19.67 |
| SDR-124 | 75.78 | 13.56 | 20.23 |
| SDR-125 | 74,02 | 10,17 | 19,66 |
| Neandertal mean, SD | 69.58, 5.33 | 11.99, 2.08 | 18.86, 2.54 |
| El Sidrón left foot | 75.01 | 11.04 | 11.36 |

**Supplementary Table S5**. Metric values (mm) of the fourth metatarsal for Neandertals^40,41^, including four cases from El Sidrón and the fourth metatarsal from the left foot.

**Supplementary Fig. S9**. Linear regression for the Neandertal sample (Supplementary Table S5) calculated excluding the fourth metatarsal from the left foot (indicated by a green arrow), with the 95% prediction intervals, for the proximal articular breadth (A) and height (B).

SD-908 shows a reduction in volume affecting the plantar half of the cuboid. There is no tuberosity on the cuboid and the plantar surface presents an irregular aspect and an increase in cortical thickness (Supplementary Fig. S10A,B). The reduction in volume results in a reduction of the area of the articular facets for the third cuneiform (Supplementary Fig. S11A, see below), calcaneum (Supplementary Fig. S10E, Supplementary Fig. S12E-F), and fourth and fifth metatarsals (Supplementary Fig. S12A, see below).

**Third cuneiform**

In a morphologically normal third cuneiform, the inferior border or crest is described as round and smooth, bearing posteriorly a small tubercle^38^. This crest provides the insertion to ligaments (cueno_3_-cuboid, plantar cuneo_3_-navicular and plantar cuneo_3_-metatarsals_3,4_), the tibialis posterior tendon, oblique head of the adductor hallucis, and the lateral arm of the origin of the flexor hallucis brevis^38^. In the medial surface of the crest is located part of the facet for the lateral facet of the base of the second metatarsal. This bone presents articular facets for the second cuneiform and cuboid (medial and lateral surfaces), and for the navicular and third metatarsal (posterior and anterior surfaces). These features can be observed in SD-121, a comparative third cuneiform from El Sidrón considered morphologically normal (Supplementary Fig. S11A).

In SD-907, the inferior crest and its posterior small tubercle are absent, and the plantar surface is irregular. As a consequence, the anterior facet for the base of the second metatarsal is reduced in height, the facet for the lateral facet of the base of the second metatarsal is absent (see metatarsals above, and Supplementary Fig. S12A), and the lateral facet for the cuboid could present a slight reduction in area (see cuboid above). This flat inferior surface presents an increase in thickness of the cortical bone as can be observe in the micro-CT sections (Supplementary Fig. S11A).

**Supplementary Fig. S10**. A: Distal, proximal, lateral, medial and inferior views of SD-908 (left) and the comparative cuboid from El Sidrón SD-1033 (right). B: micro-CT sagittal and coronal sections at the level indicated by the blue arrows in A, where the increase of the cortical thickness can be observed. C: distal and proximal views of SD-909 and the comparative navicular SD-114, with a midsagittal micro-CT section of SD-909. D: inferior view of SD-1229 and the comparative talus SD-1049. E: distal view of SD-299b and the comparative calcaneum SD-2192.

**Supplementary Fig. S11**. A: Distal, proximal, lateral, medial and inferior views of SD-907 (left) and the comparative cuneiform SD-121 (right). B: micro-CT sagittal and coronal sections at the level indicated by the blue arrows in A. C: same views of SD-906 (left) and the comparative cuneiform SD-544 (right). D: micro-CT sagittal and coronal sections at the level indicated by the blue arrows in C. E: same views of SD-905 (right) and the comparative cuneiform SDR-117 (left). F: micro-CT sagittal and coronal sections at the level indicated by the blue arrows in E.

**Second cuneiform**

The inferior border or crest of the second cuneiform is described as thin and engulfed by the two other cuneiforms^38^. This crest is the insertion to ligaments (cuneo_2_-navicular and plantar cuneiform_1,2_), and the attachment to the lateral fibrous of the flexor hallucis brevis^38^. The articular facets for the first and third cuneiform are present in the medial and lateral surfaces respectively, whereas the posterior and anterior surfaces articulate with the navicular and second metatarsal. These features can be observed in SD-544, a morphological normally second cuneiform from El Sidrón presented for comparative purposes (Supplementary Fig. S11C).

In SD-906, the inferior crest is flat, and in the micro-CT sections an increase in thickness of the cortical bone can be observed (Supplementary Fig. S11D). The articular facet for the metatarsal presents a slight reduction in height due to the flattening of the inferior border, and the articular facet for the navicular presents a more rectangular shape (Supplementary Fig. S11C). The articular facet for the first and third cuneiforms seems not to be affected by a reduction in area (Supplementary Fig. S11C).

**First cuneiform**

The inferior surface of a typical first cuneiform is rectangular and strongly convex transversely, providing insertions to the plantar cuneo_1_-navicular, plantar intercuneiform_1,2_, cuneo_1_-metatarsal_1_ and cuneo_1_-metatarsal_2,3_ ligaments, and to the peroneus longus tendon^38^. The articular facets for the first metatarsal, navicular, second cuneiform and second metatarsal are located in the distal, proximal and lateral (second cuneiform and second metatarsal) surfaces respectively. These features can be observed in SDR-117, a first cuneiform from El Sidrón presented for comparative purposes (Supplementary Fig. S11E).

**Supplementary Fig. S12**. The articular facets of the metatarsals (A), cuneiforms and cuboid (B-E), navicular (D) and calcaneum (F) are shown in a sequence from distal to proximal to display the complementary, organized reduction in the area of these facets from the tarso-metatarsal to the calcaneo-cuboid joints.

The size of SD-905 seems reduced in comparison with the size of the other tarsals of this left foot. The surface of the foot presents an irregular texture and lacks the clear convexity of the plantar surface. No increase in cortical thickness is observed (Supplementary Fig. S11F).

**Navicular**

The navicular presents in its medial end a bony prominence, the navicular tuberosity^38^, which is variable in shape and can be separated from the main bone^42^. The first plantar cuneonavicular and medial cuneonavicular ligaments arise from this tubercle, which is also where the tibialis posterior tendon inserts^38^. The inferior calcaneonavicular, plantar cubonavicular, and second and third plantar cuneonavicular ligaments are inserted to the inferior surface of this bone. The navicular presents three facets for the three cuneiforms in its anterior surface, and a concave surface for the head of the talus in its posterior surface. These features can be observed in the comparative, morphologically normal, left navicular from El Sidrón SD-114, shown for comparative purposes (Supplementary Fig. S10C).

In SD-909, the navicular tuberosity presents an abnormal shape and the beak of the navicular presents a protuberance (Supplementary Fig. S10C). In comparison with SD-114, of similar size, the area of the articular facets for the cuneiforms seems to be reduced in the plantar aspect, while the articular surface for the talus seems not to be affected by this reduction. The inferior surface of SD-909 is irregular, which is demonstrated in the sagittal micro-CT section (Supplementary Fig. S10C).

**Talus and calcaneum**

The talus and calcaneum, SD-1229 and SD-299b, from this left foot are the bones most affected by taphonomic alterations. From the comparison with other normal bones from El Sidrón (SD-1049 and SD-2192), the most obvious anomaly is the reduction in the area of the articular facets from the talocalcaneal joint (Supplementary Fig. S10D), and in the area of the articular facets for the calcaneocuboid joint (Supplementary Fig. S10E and Supplementary Fig. S10A).

**Summary**

A reduction in the area of the articular facets that form the tarsometatarsal, cuneonavicular, calcaneocuboid and talocalcaneal joints is observed. This reduction is located in the plantar surface of the bone, and the plantar borders delimitating the reduced facets are well-defined, continuous, with rounded edges and without abrupt interruptions, except for the plantar border of the lateral half of the calcaneocuboid joint, which is more irregular. This complementary, organized reduction of the plantar border of the articular facets from the tarsometatarsal to the talocalcaneal joint (Supplementary Fig. S12) would be less consistent with an antemortem trauma or a past episode of infection, where a more disorganized and irregular reduction of the plantar border of the articular facets would be expected.

The most patent alterations affect the third cuneiform, with absence of its inferior crest (Supplementary Fig. S11A), the cuboid, with a reduction in size affecting its plantar half (especially in the lateral aspect) (Supplementary Fig. S10A), and the navicular, with an abnormal shape of its tuberosity and beak (Supplementary Fig. S10C). These changes would affect the soft tissue structures associated to the plantar surface of these bones, described in detail above. For instance, in the plantar surface of these three bones there are attachments for the peroneus longus tendon, whose muscle is involved in eversion and plantar flexion of the foot, and the tibialis posterior tendon, whose muscle is involved in the inversion and plantar flexion of the foot^38,39^. The changes affecting the bones of this left foot and the associated soft tissues could have resulted in a change of the normal load pattern of the left leg of this Neandertal, and this would be connected with the increase in cortical thickness observed in the plantar surface of the cuboid (Supplementary Fig. S10B), third and second cuneiforms (Supplementary Fig. S11B,D) and possibly the calcaneum. Together, these observations are consistent with a congenital anomaly affecting the left foot.

The

coalition has affected the morphology of the plantar area by causing additional bone to build up as a base beneath the coalition. Such

changes are common in coalitions that occur outside the conﬁnes of a joint space. The lesions are smooth at the edges and pitted toward

the center, a common pattern seen in many forms of tarsal coalition

The

coalition has affected the morphology of the plantar area by causing additional bone to build up as a base beneath the coalition. Such

changes are common in coalitions that occur outside the conﬁnes of a joint space. The lesions are smooth at the edges and pitted toward

the center, a common pattern seen in many forms of tarsal coalition

**SI10. CUBOID-NAVICULAR COALITION**

As explained elsewhere^43^, the cuboid-navicular coalition is extra-articular, a fact that simplifies its identification. As shown by SD-2000 and a modern human case (Supplementary Fig. S13), the coalition cause additional bone to build up in the form of a square corner in the plantar surface of the bone. The facet presents rounded edges and pitting in the inner area. These features are diagnostic of the non-osseous coalition.

**Supplementary Fig. S13**. A: SD-2000 is shown together with SDR-114, another left navicular from El Sidrón, in distal (left) and plantar (right) views. The square contour at the plantar corner is indicated by the red arrow in SD-2000. As can be seen on the plantar view, the surface of this corner presents the facet for the cuboid. B: an archaeological case of cuboid-navicular non osseous coalition. On the left, the red arrow indicates the same feature observed on SD-2000. On the right, the complementary facet in both the navicular and the cuboid are indicated by the red triangles.

**References**

1 Pękala, P. A. *et al.* Presence of a foramen arcuale as a possible cause for headaches and migraine: Systematic review and meta-analysis. *J. Clin. Neurosci.* **54**, 113-118 (2018).

2 Ríos, L. *et al.* Acute headache attributed to whiplash in arcuate foramen and non-arcuate foramen subjects. *Eur. Spine J.* **26**, 1262-1265 (2017).

3 Ríos, L. *et al.* Shape change in the atlas with congenital midline non-union of its posterior arch: a morphometric geometric study. *Spine J.* **17**, 1523-1528 (2017).

4 Barnes, E. *Atlas of developmental field anomalies of the human skeleton: a paleopathology perspective*. (John Wiley & Sons, 2012).

5 Appleby, J., Thomas, R. & Buikstra, J. Increasing confidence in paleopathological diagnosis - Application of the Istanbul terminological framework. *Int. J. Paleopathol.* **8**, 19-21 (2015).

6 Ríos, L. *et al.* Possible further evidence of low genetic diversity in the El Sidrón (Asturias, Spain) Neandertal Group: congenital clefts of the atlas. *Plos One* **10**, e0136550 (2015).

7 Maddux, S. D., Butaric, L. N., Yokley, T. R. & Franciscus, R. G. Ecogeographic variation across morphofunctional units of the human nose. *Am. J. Phys. Anthropol.* **162**, 103-119 (2017).

8 Franciscus, R. G. Neandertal nasal structures and upper respiratory tract "specialization". *PNAS* **96**, 1805-1809 (1999).

9 Brown, O. E., Myer, C. M. & Manning, S. C. Congenital nasal pyriform apertura stenosis. *Laryngoscope* **99**, 86-91 (1989).

10 Lin, K. L. *et al.* The natural course of congenital nasal pyriform aperture stenosis. *Laryngoscope* **126**, 2399-2402 (2016).

11 Moreddu, E., Le Treut-Gay, C., Triglia, J. M. & Nicollas, R. Congenital nasal pyriform aperture stenosis: Elaboration of a management algorithm from 25 years of experience. *Int. J. Pediatr. Otorhinolaryngol.* **83**, 7-11 (2016).

12 Hengerer, A. S., Brickman, T. M. & Jeyakumar, A. Choanal atresia: embryologic analysis and evolution of treatment, a 30-year experience. *Laryngoscope* **118**, 862-866 (2008).

13 Bogusiak, K., Puch, A. & Arkuszewski, P. Goldenhar syndrome: current perspectives. *World J. Pediatr.* **13**, 405-415 (2017).

14 Kakutani, H. *et al.* Evaluation of the maxillofacial morphological characteristics of Apert syndrome infants. *Congenit. Anom.* **57**, 15-23 (2017).

15 Paradowska, A., Znamirowska-Bajowska, A. & Szelag, J. Facial Features in Binder's Syndrome - Review of the Literature. *Adv. Clin. Exp. Med.* **19**, 765-769 (2010).

16 Taitz, C., Nathan, H. & Arensburg, B. Anatomical observations of foramina transversaria. *J. Neurol. Neurosurg. Psychiatry* **41**, 170-176 (1978).

17 Abou Madawi, A., Solanki, G., Casey, A. T. & Crockard, H. A. Variation of the groove in the axis vertebra for the vertebral artery. Implications for instrumentation. *J. Bone Joint Surg. Br.* **79**, 820-823 (1997).

18 Kim, C. *et al.* A Quantitative Comparison of the Vertebral Artery and Transverse Foramen Using CT Angiography. *J. Clin. Neurosci.* **8**, 259-264 (2012).

19 Plochocki, J. H. Bilateral variation in limb articular surface dimensions. *Am. J. Hum. Biol.* **16**, 328-333 (2004).

20 Cacciola, F., Phalke, U. & Goel, A. Vertebral artery in relationship to C1-C2 vertebrae: an anatomical study. *Neurol. India* **52**, 178-184 (2004).

21 Tomasino, A. *et al.* The vertebral artery and the cervical pedicle: morphometric analysis of a critical neighborhood. *J. Neurosurg. Spine* **13**, 52-60 (2010).

22 Kim, C., Sohn, J.-H. & Choi, H.-C. Are the anomalous vertebral arteries more hypoplastic?: retrospective linear mixed model approach. *BMC Neurology* **17**, 168, doi: 10.1186/s12883-017-0951-x. (2017).

23 Ozpinar, A., Magill, S. T., Davies, J. M. & McDermott, M. W. Vertebral Artery Fenestration. *Cureus* **7**, e245 (2015).

24 Salunke, P., Sahoo, S. K. & Ghuman, M. S. Bilateral inverted vertebral arteries (V3 segment) in a case of congenital atlantoaxial dislocation: Distinct entity or a lateral variant of persistent first intersegmental artery? *Surg. Neurol. Int.* **5**, 82, doi: 10.4103/2152-7806.133642 (2014).

25 Gómez-Olivencia, A., Been, E., Arsuaga, J. L. & Stock, J. T. The Neandertal vertebral column 1: The cervical spine. *J. Hum. Evol.* **64**, 608-630 (2013).

26 Gomez-Olivencia, A. *et al.* Metric and morphological study of the upper cervical spine from the Sima de los Huesos site (Sierra de Atapuerca, Burgos, Spain). *J. Hum. Evol.* **53**, 6-25 (2007).

27 Stevens, C. A., Pearce, R. G. & Burton, E. M. Familial odontoid hypoplasia. *Am. J. Med. Genet. A* **149**, 1290-1292 (2009).

28 Wood-Jones, F. *The Principles of Anatomy: As Seen in the Hand*. (Baillière Tindall and Cox, 1944).

29 Tocheri, M. W. *Three-dimensional riddles of the radial wrist: derived carpal and carpometacarpal joint morphology in the genus Homo and the implications for understanding the evolution of stone tool-related behaviors in hominins*, Arizona State University, (2007).

30 Trinkaus, E. *The Krapina Human Postcranial Remains: Morphology, Morphometrics and Paleopathology*. (Faculty of Humanities and Social Sciences, University of Zagreb, 2016).

31 Louis, D. S., Calhoun, T. P., Garn, S., Carroll, R. E. & Burdi, A. Congenital bipartite scaphoid--fact or fiction? *JBJS* **58**, 1108-1112 (1976).

32 Chang, A. C., Leonello, D. T. & Webb, J. M. Congenital bipartite scaphoid. *J. Hand Surg. Eur.* **40**, 537-538 (2015).

33 Doman, A. N. & Marcus, N. W. Congenital bipartite scaphoid. *J. Hand Surg. Am.* **15**, 869-873 (1990).

34 Dubrana, F. *et al.* Bilateral bipartite carpal scaphoid bone: congenital or non union origin? *Rev. Chir. Orthop. Reparatrice Appar. Mot.* **85**, 503-506 (1999).

35 Takemitsu, Y., Nakayama, Y., Ota, H., Matsumoto, Y. & Kida, H. Bilateral bipartite carpal scaphoid: a case report and literature review. *Hand Surg.* **19**, 427-431 (2014).

36 Trinkaus, R. Human patellar articular proportions: recent and Pleistocene patterns. *J. Anat.* **196**, 473-483 (2000).

37 Hoechel, S., Schulz, G. & Mueller-Gerbl, M. Insight into the 3D-trabecular architecture of the human patella. *Ann. Anat.* **200**, 98-104 (2015).

38 Kelikian, A. S. *Sarrafian's anatomy of the foot and ankle: Descriptive, topographic, functional: Third edition*. (2012).

39 Bannister, L. H. *Gray's anatomy*. (Churchill Livingstone, 1999).

40 Courtaud, P. Deux os du pied provenant des niveaux moustériens de la grotte de Kébara (Israël). *Bull. Mem. Soc. Anthropol. Paris* **1**, 45-58 (1989).

41 Pablos, A. *et al.* New foot remains from the Gran Dolina-TD6 Early Pleistocene site (Sierra de Atapuerca, Burgos, Spain). *J. Hum. Evol.* **63**, 610-623 (2012).

42 Offenbecker, A. M. & Case, D. T. Accessory navicular: A heritable accessory bone of the human foot. *Int. J. Osteoarchaeol.* **22**, 158-167 (2012).

43 Case, D. T. & Burnett, S. E. Identification of tarsal coalition and frequency estimates from skeletal samples. *Int. J. Osteoarchaeol.* **22**, 667-684 (2012).

1. Adapted from “Nomenclature in Paleopathology,” by Keith Manchester, Alan Ogden and Rebecca Storm, Paleopathology Newsletter No. 175, September2016. See https://paleopathologyassociation.wildapricot.org/Nomenclature-in-Paleopathology. [↑](#footnote-ref-2)
2. The formula used by Plochocky (*Asym=((2x(R-L))/(R+L))x100*) standardizes the asymmetry to within-individual percentages. Negative values indicates that the left side is larger and a positive value that the right side is larger, and the further the value from zero in either direction, the greater the asymmetry. The formula for absolute asymmetry was provided by Asier Gómez-Olivencia (*Asym=((R-L)/smaller)x100*). [↑](#footnote-ref-3)
